# Retrospective investigation about anesthetic management of biliary atresia in children: laparoscopic versus conventional Kasai portoenterostomy

**DOI:** 10.1186/s40981-019-0228-z

**Published:** 2019-02-07

**Authors:** Takehito Sato, Kimitoshi Nishiwaki

**Affiliations:** Department of Anesthesiology, Nagoya University Graduate School of Medicine, 65 Tsurumai-cho, Showa-ku, Nagoya city, Aichi 466-8550 Japan

**Keywords:** Kasai portoenterostomy, Biliary atresia, Laparoscopy, Infant, Hypothermia

## Abstract

**Purpose:**

Biliary atresia can be fatal if surgery is not performed early. Laparoscopic Kasai portoenterostomy was recently introduced in our hospital. Despite laparoscopic surgery generally provides advantages, there are few studies of laparoscopic surgery performed in infants.

We retrospectively compared anesthesia management of patients undergoing laparoscopic Kasai portoenterostomy and conventional Kasai portoenterostomy and investigated anesthetic complications of laparoscopic Kasai portoenterostomy.

**Methods:**

Fifty-three biliary atresia patients who underwent surgery from April 2010 to September 2017 were assessed: 28 who underwent laparoscopy (L group) and 25 who underwent laparotomy (O group) were included. We compared body temperature, cases of hypothermia, the lowest mean blood pressure, bleeding volume, infusion volume and urine volume (ml/kg and ml/kg/h), age, weight, operation time, and the number of patients postoperatively admitted to the intensive care unit.

**Results:**

In the L group, volume of bleeding was significantly smaller, and the lowest body temperature was significantly lower in the L group than in the O group (22 ± 35.1 mL vs 70 ± 34.5 mL, respectively, *P* < 0.01; 35.6 ± 0.8 °C vs 36.5 ± 0.4 °C, respectively, *P* < 0.01). And severe hypothermia was significantly more in the L group (7 cases vs 0 cases *P* = 0.01). There was an inverse correlation between the lowest body temperature and anesthesia time (*r* = − 0.464, *P* < 0.01). Multiple linear regression analysis revealed that anesthesia time was a significant predictor of hypothermia.

**Conclusion:**

Our study revealed that laparoscopic surgery in infants reduced bleeding, but induced hypothermia and upper airway edema may be caused by relatively excessive infusion. At laparoscopic Kasai surgery, anesthesiologist is recommended to prevent hypothermia and need to pay attention to amount of infusion.

**Trial registration:**

This study was approved by the Ethics Committee of Nagoya University (2017-0290) and registered with the UMIN Clinical Trial Registry (UMIN000033158).

## Introduction

Biliary atresia is characterized by high choler-related liver damage as a result of inherent biliary tract occludes. It has an incidence of 1 in 9000–17,000 newborns [1] and a high fatality rate if surgery is not performed at an early stage [2, 3]. Biliary atresia can be radically treated by performing “Kasai operation” (Kasai portoenterostomy) [4]. Kasai portoenterostomy is widely performed and results in improved liver prognosis in approximately 60% of biliary atresia patients [3].

Kasai portoenterostomy is commonly performed by laparotomy (conventional open surgery); however, recent improvements in laparoscopic devices have enabled the Kasai operation to be performed under laparoscopy. There are various advantages and disadvantages of laparoscopic surgery. There are few reports regarding anesthesia management during the laparoscopic procedure. Furthermore, no studies have examined anesthesia management at a single facility because of the limited number of biliary atresia patients in this country.

In the present study, we retrospectively investigated the anesthesia management of patients undergoing laparoscopic Kasai portoenterostomy and those undergoing conventional Kasai portoenterostomy at a single institution and investigated complication of laparoscopic Kasai portoenterostomy, especially intraoperative hypothermia.

## Materials and methods

This study was approved by the Ethics Committee of Nagoya University (approval number, 2017-0290) and registered with the UMIN clinical Trial Registry (UMIN 000033158). Eighty-five patients scheduled to undergo elective Kasai operation between April 2010 and September 2017 were assessed. Inclusion criteria were (1) American Society of Anesthesiologists classification of I–III, (2) being 0–90 days after birth, and (3) absence of severe congenital heart disease (which required surgical treatment such as single ventricle.). Exclusion criteria were (1) reoperation (including revision operation and hemostasis), (2) the patient was diagnosed as not having biliary atresia, and (3) informed consent not provided by the patient’s family.

Finally, 53 patients were enrolled after excluding 32 patients: 25 underwent conventional Kasai portoenterostomy surgery (O group) and 28 underwent laparoscopic Kasai portoenterostomy (L group).

The primary outcome was the cases and degree of hypothermia. Secondary outcomes included amount of bleeding and cannot do extubation after operation between two groups.

Patient information was collected from the Anesthesia Record System, and patients in the two groups were compared for age (day), body weight (kg), sex, management of anesthesia (induction, drug, and type of tracheal intubation tube), operation time (min), lowest arterial pressure (mmHg), total urine volume (ml) and intraoperative average urine flow rate (ml/kg/h), infusion volume (ml) and intraoperative average infusion rate (ml/kg/h), transfusion (case), bleeding volume (ml), lowest body temperature (°C), upper airway edema (case), intraoperative complications: hypothermia (mild hypothermia: body temperature < 36.0 °C, and severe hypothermia < 35.0 °C as previously described [5]), cannot extubate after surgery, mass bleeding (volume of bleeding > 150 ml [6]), cardiac arrest, severe hypotension (systolic blood pressure < 50 mmHg), hypoxia (SpO_2_ < 89%), and hypercapnia (EtCO_2_ > 70 mmHg) [5–7].

All data are presented as mean ± standard deviation and were analyzed using the chi-square test and Mann–Whitney *U* test. A liner regression was applied to examine the bivariate regression between the primary outcome (body temperature) and other variables, and to investigate the independent variables which affected the body temperature, multiple regression was applied. A *P* value of < 0.05 was considered statically significant. Statcel4 (OMS Publishing Inc., Saitama, Japan) and EZR (Saitama Medical Center, Jichi Medical University, Saitama, Japan) [8], R (version 3.3.1, 2016-06-21, svn rev 70800, Vienna, Austria), and SPSS® 25.0 for Windows® (SPSS Inc., Chicago, IL, USA) were used for all statistical analyses.

## Results

No patients received any premedication. There were no significant differences in age and body weight between the two groups. Both groups contained more girls than boys (Table 1), which is consistent with the statistics of biliary atresia. All patients received general anesthesia, and there was no significant difference between the two groups.

**Table 1:** Characteristics of patients

	O group *n* = 25	L group *n* = 28
Age (days)	59 ± 8.	55 ± 14
Height (cm)	53.7 ± 4	54.5 ± 4
Weight (kg)	4.1 ± 0.9	4.2 ± 0.7
Male/female	7/18	8/20
Direct bilirubin (mg/dl)	4.5 ± 1.3	4.8 ± 1.8

There were no differences between the two groups

Anesthesia time in the laparoscopy group was significantly longer than in the open group (*P* = 0.01). Patients in the laparoscopy group had a significantly lowest body temperature and smaller bleeding volume than those in the open group (*P* < 0.01 for both). And laparoscopy group had more mild hypothermia (*P* = 0.004) and severe hypothermia patients (*P* = 0.01). Four patients in the laparoscopy group were admitted to the intensive care unit without extubation because of extreme hypothermia (34.6–34.9 °C) and upper airway edema.

There was no significant difference in the volume of infusion and urine per weight per time between the two groups. Transfusion was needed in one patient in the laparoscopy group and six in the open group (*P* = 0.04) (Table 2). There were no patients with severe intraoperative complication such as mass bleeding, intraoperative hypoxia, pneumothorax, or cardiac arrest, but only one patient had severe hypercapnia at the laparoscopy group. We investigated the correlation of body temperature and time of anesthesia. The lowest body temperature was significantly correlated with anesthesia time (*r* = − 0.464, *P* < 0.001) and total fluid volume (*r* = − 0.291, *P* = 0.003), but not with age, body weight, bleeding volume, and urine volume. A multiple linear regression was calculated to predict body temperature based on the time of anesthesia and total amount of urine and infusion. The time of anesthesia was a significant predictor of hypothermia (Table 3).

**Table 2:** Intraoperative patient data

	O group *n* = 25	L group *n* = 28	*P* value
Anesthesia time (min)	224 ± 42.6	309 ± 71.4	< 0.01
Lowest mean arterial pressure (mmHg)	31 ± 2	32 ± 3	> 0.05
Total urine volume (ml)	29 ± 28.1	52.5 ± 49	< 0.01
Intraoperative average urine flow rate (ml/kg/h)	1.14 ± 1.19	1.91 ± 1.41	> 0.05
Total infusion volume (ml)	494 ± 249	580 ± 180	< 0.05
Intraoperative average infusion rate (ml/kg/h)	19.7 ± 9.25	16.6 ± 14.5	> 0.05
Transfusion (case)	6	1	< 0.05
Bleeding volume (ml)	70 ± 34.5	22 ± 35.1	< 0.01
Lowest body temperature (°C)	36.5 ± 0.4	35.6 ± 0.8	< 0.01
Mild hypothermia (case)	4	16	< 0.01
Severe hypothermia (case)	0	7	< 0.05
Upper airway edema (case)	0	4	> 0.05

**Table 3:** Result of multiple regression of body temperature and other value

Parameter	Estimate	SE	t value	*P* value
Intercept	37.7	0.43	86.9	< 0.001
Anesthesia time (min)	− 0.0007	0.001	− 3.42	0.00127**
Total fluid (ml)	− 0.0001	0.0003	− 1.91	0.06
Total urine (ml)	− 0.0004	0.002	− 0.07	0.9

**Significant parameter (*P* < 0.01)

## Discussion

We compared anesthesia management between patients undergoing laparoscopic and conventional open Kasai portoenterostomy for biliary atresia. Hypothermia was more common in patients receiving laparoscopic surgery than those receiving open surgery. Because body temperature was inversely correlated with anesthesia time and with total amount of fluid, patients receiving laparoscopic surgery developed hypothermia as a result of extended duration of surgery and pneumoperitoneum. Cold carbon dioxide gas is one of the causes of hypothermia, and anesthesiologists should prevent hypothermia by increasing the room temperature, by using a warm blanket and by warming fluid and blood for transfusion.

In our study, patients in the laparoscopy group had more hypothermia than those in the open surgery group. And there were also significantly many patients with severe hypothermia at laparoscopy group. There was a negative correlation of body temperature, anesthesia time, and total amount of fluid. As in past reports [9, 10], at Kasai portoenterostomy, hypothermia was caused by laparoscopy surgery.

Four patients were unable to be extubated in the operating room owing to upper airway edema after laparoscopic surgery. Although there were no differences in the intraoperative average fluid infusion rate, the total infusion volume in patients receiving laparoscopic surgery was significantly larger than that in those receiving open surgery. Because blood loss was significantly smaller during laparoscopic than open surgery, we may have infused excessive fluid in patients receiving laparoscopic surgery, resulting in upper airway edema. There is also a risk of excessive fluid infusion as a result of reduced venous return by pneumoperitoneum, leading to oliguria, and intraoperative infusion volume should be reduced during laparoscopic Kasai operation [9, 10].

Surgery provides a radical cure for biliary atresia, whereby Kasai operation or liver transplantation is required within 60 days after birth for survival. Liver transplantation from brain-dead donors is rarely performed in Japan; rather, Kasai operation is commonly performed for biliary atresia. Since the first case report of laparoscopic Kasai operation for biliary atresia by Esteves et al. [11] in 2002, it has been performed in various countries [12, 13]. However, postoperative liver prognosis is poorer following laparoscopic Kasai operation than following conventional laparotomy [12–14]. According to a meta-analysis by Lishuang et al. [12], laparoscopic Kasai operation has not replaced open surgery.

Despite these limitations, laparoscopic surgery provides various advantages such as minimum surgical wound and reduced postoperative pain (Table 4). In addition, a new surgical technique of laparoscopic Kasai operation has recently been reported [15]. Hence, laparoscopic Kasai operation will provide greater medical adaption.

**Table 4:** Advantages and disadvantages of laparoscopic Kasai surgery

Advantages	Disadvantages
Minimized operative wound	Pneumoperitoneum
Little postoperative pain	Hypercapnia
Few cosmetic problems	Low body temperature
Reduced bleeding	Insufficient ventilation
Earlier postoperative recovery	Bronchial intubation
Able to share the operative view with other doctors	Increased operation time
	Requires an advanced operation technique
	Does not provide evidence for liver prognosis

There are several limitations to this study: the limited number of patients and the retrospective study design. In Japan, there were approximately cases of 115 biliary atresia patients in 2015 [16]. Hence, it is difficult to include many patients. While our facility is eminent in this country enabling us to report the Kasai operation at a single institution, cases of more patients are required. In addition, the anesthesia method and infusion volume were not unified in this study. Prospective study addressing these issues is expected in the future.

To conclude, we reported and compared the anesthesia management of patients undergoing laparoscopic Kasai portoenterostomy and those undergoing open Kasai portoenterostomy. During laparoscopic Kasai surgery, we should administer less amount of fluid for avoiding excessive infusion. As a characteristic of laparoscopic surgery in infants, reduced bleeding and hypothermia influenced by pneumoperitoneum are important. Active warming following entry into the operation room is recommended to prevent hypothermia.

## Abbreviations

ICU: Intensive care unit
L group: Laparoscopic Kasai group
O group: Open Kasai (conventional) group
